# Frequencies and characteristics of genome-wide recombination in *Streptococcus agalactiae*, *Streptococcus pyogenes*, and *Streptococcus suis*

**DOI:** 10.1038/s41598-022-04995-5

**Published:** 2022-01-27

**Authors:** Isaiah Paolo A. Lee, Cheryl P. Andam

**Affiliations:** 1 University of New Hampshire, Durham, NH 03824 USA; 2 University at Albany, State University of New York, New York, 12222 USA

**Keywords:** Bacteriology, Molecular evolution, Microbiology

## Abstract

*Streptococcus* consists of ecologically diverse species, some of which are important pathogens of humans and animals. We sought to quantify and compare the frequencies and characteristics of within-species recombination in the pan-genomes of *Streptococcus agalactiae, Streptococcus pyogenes* and *Streptococcus suis*. We used 1081, 1813 and 1204 publicly available genome sequences of each species, respectively. Based on their core genomes, *S. agalactiae* had the highest relative rate of recombination to mutation (11.5743) compared to *S. pyogenes* (1.03) and *S. suis* (0.57). The proportion of the species pan-genome that have had a history of recombination was 12.85%, 24.18% and 20.50% of the pan-genomes of each species, respectively. The composition of recombining genes varied among the three species, and some of the most frequently recombining genes are implicated in adhesion, colonization, oxidative stress response and biofilm formation. For each species, a total of 22.75%, 29.28% and 18.75% of the recombining genes were associated with prophages. The cargo genes of integrative conjugative elements and integrative and mobilizable elements contained genes associated with antimicrobial resistance and virulence. Homologous recombination and mobilizable pan-genomes enable the creation of novel combinations of genes and sequence variants, and the potential for high-risk clones to emerge.

## Introduction

The Gram-positive genus *Streptococcus* (phylum Firmicutes) comprises 188 recognized species (https://lpsn.dsmz.de/search?word=streptococcus as of July 12, 2021; List of Prokaryotic names with Standing in Nomenclature (LPSN)^1^). It consists of diverse bacteria that display a wide breadth of ecological interactions with their eukaryotic hosts, from commensals to pathogens and with broad or restricted host ranges^2^. The most well-known is *Streptococcus pneumoniae* (or pneumococcus), which is a common resident of the upper respiratory tract of humans^3^. It is also a major cause of otitis media and pneumonia, as well as invasive infections such as bacteremia and meningitis^3,4^. Other *Streptococcus* species are equally notable. *Streptococcus agalactiae* (also known as Group B *Streptococcus* or GBS) is often found in normal microbiota of the gastrointestinal and genitourinary tracts of healthy women^5,6^. It is the leading cause of neonatal sepsis and meningitis, and can also lead to chronic neurologic sequelae such as seizures, cognitive impairment and motor deficits^5,6^. *S. agalactiae* also causes mastitis in cattle^7,8^ and has also been reported in diverse animals^9^. *Streptococcus pyogenes* (also known as Group A *Streptococcus* or GAS) colonizes the epithelial surface, primarily on the skin and nasopharynx of humans^10,11^. It causes a wide range of suppurative diseases (e.g., impetigo, necrotizing fasciitis), non-suppurative diseases (e.g., acute rheumatic fever, acute glomerulonephritis) and toxin-mediated diseases (e.g., scarlet fever, toxic shock syndrome)^10,11^. *Streptococcus suis* causes a wide range of infections in pigs, such as meningitis, arthritis and sepsis^12^. It is carried asymptomatically by healthy pigs on their tonsils, nasal cavities and gastrointestinal tract^12^. Animal-to-human transmission of *S. suis* is not uncommon among humans who work in the swine industry or those who consume raw pork products^13,14^.

Recombination greatly contributes to the exceptional genetic and phenotypic variation among members of a *Streptococcus* species^15,16^. Frequent or large-scale recombination events can result in the emergence of novel genotypes with characteristics that are unpredictable. For example, recombination in the capsular locus and antibiotic resistance genes of *S. pneumoniae* enable lineages to successfully evade the impacts of selective pressures such as vaccination and antibiotic treatment^17^. It also contributes to the successful adaptation and switching to new eukaryotic hosts^9^. Most studies on genetic recombination from a population genomic standpoint in this genus have focused on the highly transformable and hyper-recombining *S. pneumoniae* (for examples, see references^17,18^). Other *Streptococcus* species are also known to recombine. *S. agalactiae* exhibits variable amounts of recombination, with the propensity for recombination being strain-dependent^19^. *S. pyogenes* undergoes widespread recombination, resulting in the high genome plasticity found in strains worldwide^20^. While recombination in *S. agalactiae*^9,21,22^*, S. pyogenes*^23,24^ and *S. suis*^25^ have been previously documented, systematic comparisons of recombination between these species is limited.

Here, we sought to quantify and compare the frequencies and characteristics of within-species recombination in the pan-genomes of *S. agalactiae, S. pyogenes* and *S. suis*. We used 1081, 1813 and 1204 publicly available genome sequences of each species, respectively. We also surveyed those recombining genes to determine the contributions of mobile genetic elements in their mobilization. Our study highlights the remarkable ability of each *Streptococcus* species to continually create new combinations of genes and sequence variants that selection can later act on. Understanding the contributions of recombination and a mobilizable pan-genome to their genomic evolution will aid in tracking public health threats posed by newly emerging, high-risk lineages.

## Results

### Pan-genome and phylogenetic structure within species

**Figure 1:**
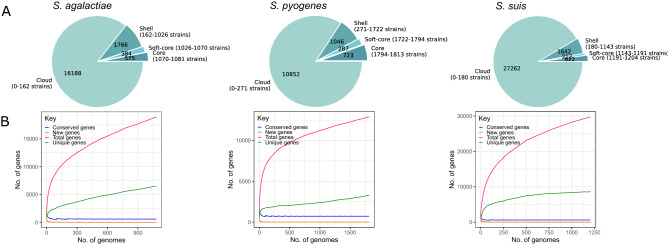
Pan-genome characteristics of each *Streptococcus* species calculated using Roary. (**a**) Pie charts showing the distribution of core, soft core, shell and cloud genes. (**b**) Accumulation curves showing the size of the pan-genome, i.e., the totality of unique genes present in each species (pink line), the size of the core genome, i.e., genes that are present in at least 99% of the strains (blue line), the number of unique genes, i.e., genes unique to an individual strain (green line), and new genes, i.e., genes not found in the previously compared genomes (orange line) in relation to numbers of genomes being compared. Detailed results of Roary are shown in Supplementary Table S2.

We used 1081, 1813 and 1204 high quality genome sequences of *S. agalactiae, S. pyogenes* and *S. suis,* respectively, downloaded from the RefSeq database^26^ of the National Center for Biotechnology Information (NCBI) (Supplementary Table S1). Genome sizes of *S. agalactiae, S. pyogenes* and *S. suis* ranged from 1.79 to 2.39 Mb (mean = 2.08 Mb), 1.67 to 1.99 Mb (mean = 1.79 Mb) and 1.71 to 2.72 Mb (mean = 2.15 Mb), respectively. The number of predicted genes per genome ranged from 1815–2375 (mean = 2044) in *S. agalactiae*, 1549–2040 (mean = 1712) in *S. pyogenes* and 1580–2561 (mean = 2044) in *S. suis* (Supplementary Table S1). The pan-genome of *S. agalactiae* consisted of 18,913 orthologous gene clusters (Fig. 1A; Supplementary Table S2), of which 575 genes were core genes, 384 were soft core genes, 1766 were shell genes and 16,188 were cloud genes. The pan-genome of *S. pyogenes* consisted of 12,908 orthologous gene clusters (Fig. 1A; Supplementary Table S2), of which 723 genes were core genes, 287 were soft core genes, 1046 were shell genes and 10,852 were cloud genes. Lastly, the pan-genome of *S. suis* consisted of 29,738 orthologous gene clusters (Fig. 1A; Supplementary Table S2), of which 622 genes were core genes, 212 were soft core genes, 1642 were shell genes and 27,262 were cloud genes.

The size of the pan-genome and its increase or decrease in size upon addition of new strains can be used to predict the future rate of discovery of novel genes in a species^27^. In all three species, we found that their pan-genomes increased with the addition of new genomes, while the core genome decreased and began to plateau at approximately 50, 80 and 30 genomes in *S. agalactiae, S. pyogenes* and *S. suis*, respectively (Fig. 1B). The number of unique genes that have been observed exactly once also continued to increase as each genome is added. Their pan-genomes were dominated by genes found in one or few strains. In all, these results indicate that the pan-genomes of the three species are large and open, i.e., the size of the pan-genome is increasing and unbounded by the number of genomes considered^27^.

**Figure 2:**
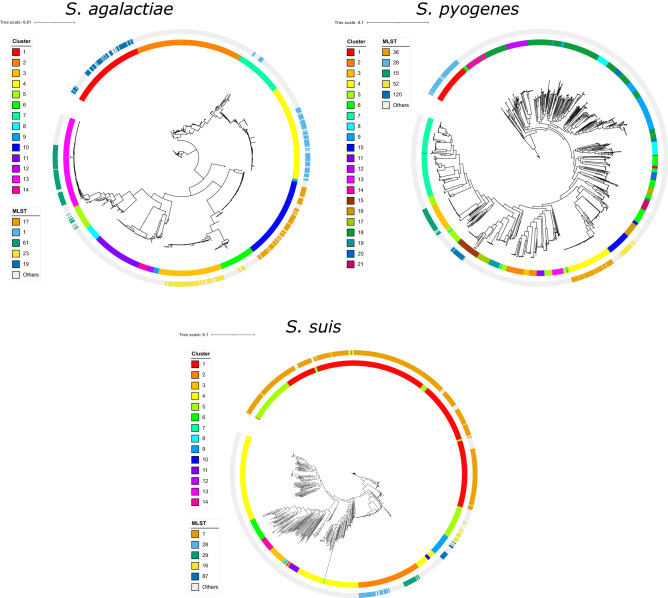
Midpoint-rooted maximum likelihood trees of each *Streptococcus* species. Scale bar represents nucleotide substitutions per site. BAPS clusters and sequence types (STs) are shown on outer rings. For visual clarity, only the most common STs are highlighted with color. Detailed multilocus ST results are listed in Supplementary Table S1.

We next examined the phylogenetic diversity and population structure within each species. We used the Bayesian hierarchical clustering program RhierBAPS^28^ to reveal sequence clusters that comprised each species. Sequence clusters are groups of related strains with similar or closely related genotypes^29^. Multilocus sequence typing (MLST) revealed a total of 96, 435 and 211 previously known sequence types (STs) in *S. agalactiae, S. pyogenes* and *S. suis*, respectively (Fig. 2; Supplementary Table S1). The most common STs in *S. agalactiae* were ST 17 (96 genomes), ST 1 (93 genomes), ST 61 (91 genomes), ST 23 (82 genomes) and ST 19 (67 genomes). The most common STs in *S. pyogenes* were ST 36 (100 genomes), ST 28 (96 genomes), ST 15 (64 genomes), ST 52 (38 genomes) and ST 120 (36 genomes). The most common STs in *S. suis* were ST 1 (523 genomes), ST 28 (45 genomes), ST 29 (26 genomes), ST 16 (20 genomes) and ST 87 (17 genomes).

### Quantification of homologous recombination in species pan-genomes

For each species, we sought to estimate six evolutionary and recombination parameters using the correlation profiles of synonymous substitutions for pairs of homologous sequences in the core genome alignment. We used the program mcorr to estimate these parameters^30^*.* The three species varied in terms of the six parameters (Table 1, Supplementary Table S3). The diversity (d), which is generated from both recombination and the accumulation of mutations of the clonal lineage^30^, was highest in *S. suis* (0.0417) compared to *S. agalactiae* (0.0072) and *S. pyogenes* (0.0096). The mean number of mutations per locus since divergence of a pair of homologous sites (or mutational divergence [θ])^30^ is highest in *S. suis* (0.151) compared to *S. agalactiae* (0.0165) and *S. pyogenes* (0.0560). Recombinational divergence (ϕ) was highest in *S. agalactiae* (0.1905) compared to that of *S. pyogenes* (0.0575) and *S. suis* (0.865). We next calculated the ratio ϕ/θ, which gives the relative rate of recombination to mutation^30^. The ratio ρ/θ is a measure of the frequency at which recombination occurs relative to mutation^31^. *S. agalactiae* has a much higher ϕ/θ value (11.5743) than that of *S. pyogenes* (1.0265) and *S. suis* (0.5737). This means that in *S. agalactiae* for example, recombination events occur 11.6X as often as point mutations in the population. The mean fragment size ($\overline{{\text{f}}}$) of a recombination event was highest in *S. suis* (3147 bp) compared to *S. agalactiae* (1303 bp) and *S. pyogenes* (389 bp). Lastly, the recombination coverage (c) indicates the fraction of the genome whose diversity was derived from recombination events since its last common ancestor and ranges from 0 (indicating clonal evolution) to 1 (indicating complete recombination)^30^. We estimated this parameter to be 0.445 in *S. agalactiae*, 0.172 in *S. pyogenes* and 0.330 in *S. suis*. These values mean that 44.5%, 17.2% and 33.0% of sites in the core genome sequence of each species, respectively, originated from recombination events. For comparison, the parameters for 84 genomes of transformed *S. pneumoniae* strains^32^ calculated by the authors of mcorr were d = 6.80 × 10^–5^, θ = 0.084, ϕ = 0.110, ϕ/θ = 1.30, $\overline{{\text{f}}}$ = 560 and c = 0.09^30^. In summary, the parameters obtained for the three *Streptococcus* species were comparable, and even higher for some parameters, to those reported in the frequently recombining *S. pneumoniae* as well as in other well-known species of bacterial pathogens^30^.

**Table 1:** Evolutionary and recombination parameters calculated by mcorr.

	*S. agalactiae*	*S. pyogenes*	*S. suis*
d	0.0072	0.0096	0.0417
θ	0.0165	0.0560	0.1508
ϕ	0.1905	0.0575	0.0865
ϕ/θ	11.5743	1.0265	0.5737
$\overline{{\text{f}}}$	1302.99	389.21	3147.13
c	0.4448	0.1721	0.3298

Core genome alignment of each species was used as input in mcorr with 1000 bootstrapped replicates. Bootstrapping results are shown in Supplementary Table S3.

*d* diversity brought into the population by recombination and clonal diversity, *θ* mutational divergence, *ϕ* recombinational divergence, *ϕ/θ* relative rate of recombination to mutation, $\overline{{\text{f}}}$ mean fragment size of a recombination event, *c* recombination coverage.

**Figure 3:**
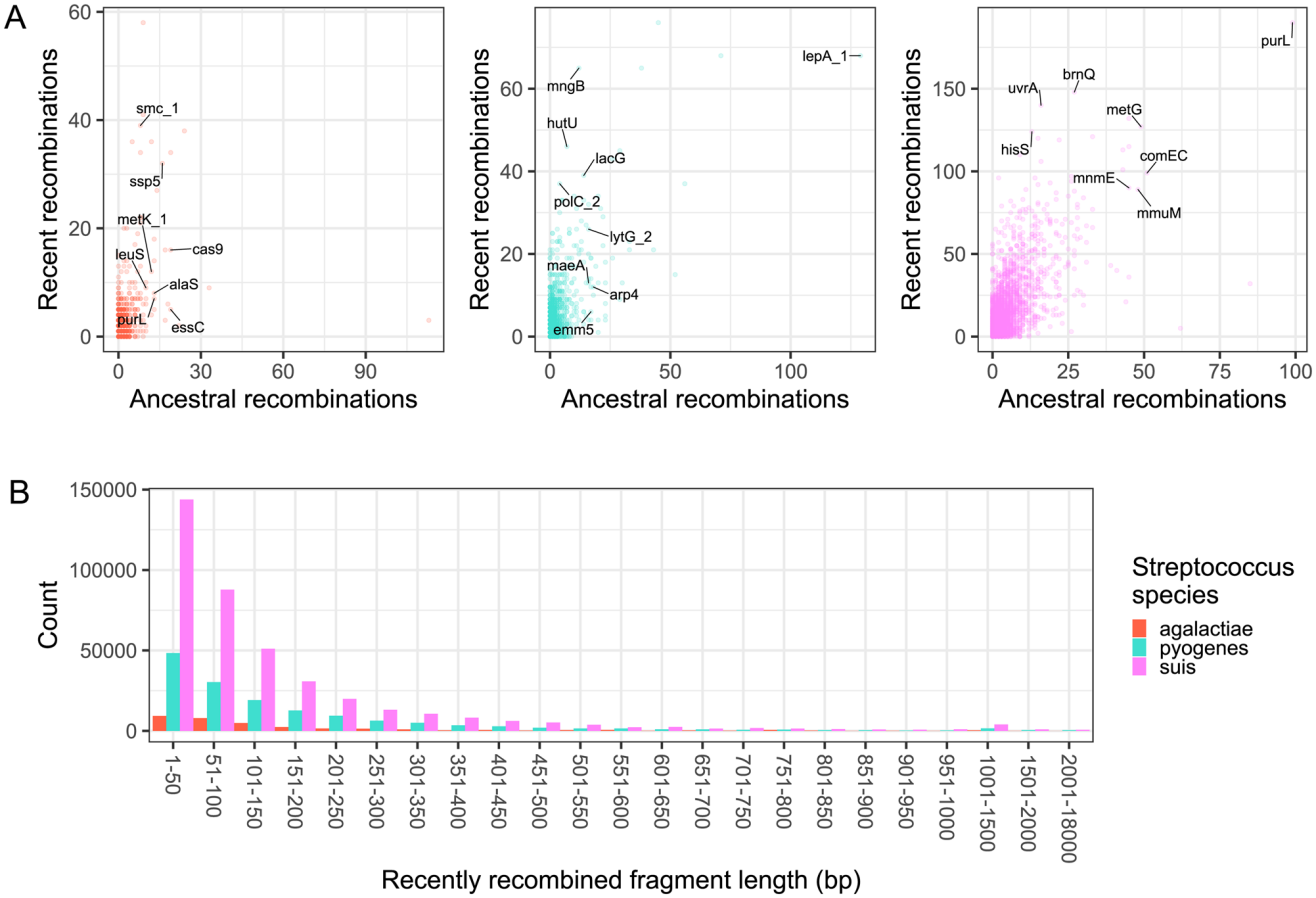
Recombining genes in the pan-genome of the three *Streptococcus* species. (**A**) Number of recent and ancestral recombination events calculated by fastGEAR in each *Streptococcus* species. For visual clarity, only some of the frequently recombining genes with known functions are labeled. (**B**) Frequency histogram comparing the sizes of recombined DNA in the three *Streptococcus* species. Only recent recombinations are shown. Detailed list of fastGEAR results is shown in Supplementary Table S4.

**Figure 4:**
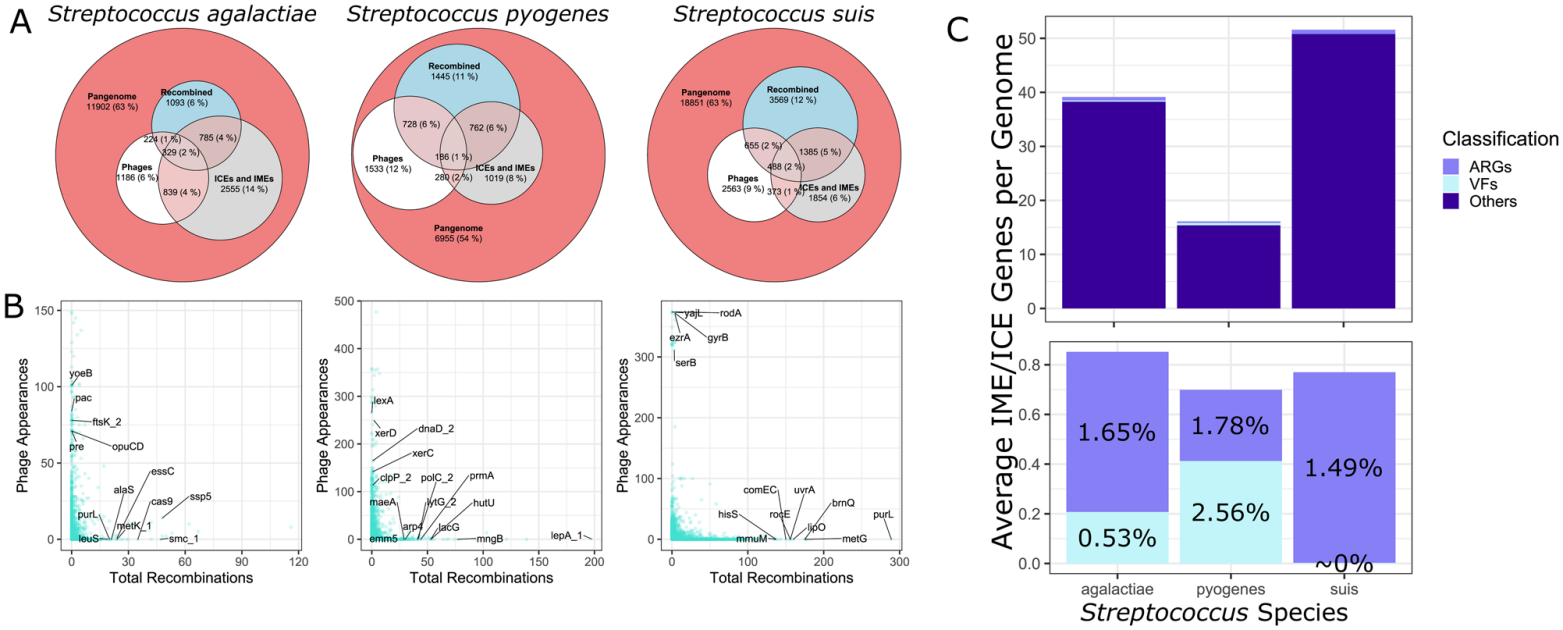
Mobile genetic elements in *Streptococcus* recombination. (**A**) Euler diagrams showing the proportions of the pan-genome composed of genes associated with phages, ICEs and IMEs, and homologous recombination events of each *Streptococcus* species. Within each diagram, the areas of the circles are proportional to the number of genes. (**B**) Total number of recombination events calculated by fastGEAR in each *Streptococcus* species of each gene plotted against the number of times each gene is detected on a phage by PhiSpy. For visual clarity, only some of the frequently recombining genes and genes frequently found on phages with known functions are labeled. Detailed list of fastGEAR results is shown in Supplementary Table S4. (**C**) Bar graphs showing the mean number of ICE/IME-associated cargo genes per genome of each *Streptococcus* species. These are grouped as antimicrobial resistance genes (ARGs), virulence factors (VFs) and other genes identified. The graph above includes all three categories, while the graph below focuses on ARGs and VFs. The percentages shown are the fraction of total ICE/IME-associated genes that comprise ARGs and VFs of each species.

We next sought to identify the specific genes in each species’ pan-genome that have had experienced recombination (Fig. 3; Supplementary Table S4). We used fastGEAR^33^ on individual sequence alignments of each core and shared accessory genes. Among those genes with known function in *S. agalactiae* (Fig. 3), the most frequently recombined gene was *smc* (structural maintenance of chromosomes), which plays an important role in chromosome organization^34^. The gene *ssp-5* codes for an agglutinin receptor and is responsible for adhesion and colonization of *Streptococcus* to different substrates inside the host^35^. The gene *cas9* codes for a CRISPR-associated endonuclease nuclease and its inactivation has been demonstrated to reduce adhesion, intracellular survival and virulence of *S. agalactiae*^36^. The reduced transcription of *cas9* has also been found to affect the expression of another frequently recombined gene *metK*^36^, which codes for methionine adenosyltransferase. In *S. agalactiae*, we found 2431 genes that had experienced recombination, which represents 12.85% of the species pan-genome (Fig. 4).

In *S. pyogenes* (Fig. 3), the most frequently recombined gene with known function is *lepA*, which is responsible for the polymerization of pili in *S. pyogenes,* which in turn plays a role in adhesion, biofilm formation and pathogenicity^37^. Another frequently recombined gene is *mngB* is involved in the transport and metabolism of the osmolyte 2-*O*-α-mannosyl-d-glycerate^38^. This compound is widely used by thermophilic prokaryotes as a protective osmolyte at high temperature and salinity^38^. *S. pyogenes* had a total of 3121 genes that had experienced recombination, which is equivalent to 24.18% of the species pan-genome (Fig. 4).

Finally, the most frequently recombined gene with known function in *S. suis* (Fig. 3) was the purine biosynthesis gene *purL*. This gene has been implicated in lung infection by *S. pneumoniae*^39^ and in biofilm formation in *Streptococcus sanguinis*^40^. The gene *brnQ* is a branched-chain amino acid transporter found in many bacterial species and its gene product plays a role in adaptation to nutrient limitation, proliferation during infection and evasion of host defenses^41^. The gene *metG* encodes the methionyl-tRNA synthetase is essential for protein synthesis, and inhibitors targeting this protein may represent an effective mechanism of action of antibiotics to target gram-positive pathogens, including *Streptococcus*^42^. The gene *uvrA* is involved in DNA repair^43^, and has been implicated in adaptive response to low pH in *Streptococcus mutans*^44^. The product of *comEC* is a major component of the bacterial transformation machinery, and functions in the binding, single strand degradation and membrane translocation of DNA^45^. *S. suis* had a total of 6097 genes that had experienced recombination, which represents 20.50% of the species pan-genome (Fig. 4).

Majority of the recombination events involve short DNA fragments less than 300 bp in size (Fig. 3B). Large recombination events (> 1000 bp) occurred less frequently. The longest recent recombination blocks for each species were 5547 bp in *S. agalactiae*, 17,772 bp in *S. pyogenes*, and 8798 bp in *S. suis*. Both micro-recombination (i.e., frequent replacement of short DNA fragments) and macro-recombination (i.e., rarer multifragment, saltational replacements) events have been reported in the highly recombining *S. pneumoniae*^46^ and we report similar findings in these three *Streptococcus* species. For comparison, macro-recombination in the highly recombining *S. pneumoniae* has been reported to reach up to 100,000 bp^47^.

### Contributions of prophages to recombination

Using PhiSpy^48^, we next determined if there is any overlap between the recombining genes identified by fastGEAR and genes associated with prophages (Supplementary Table S5). Figure 4A shows the proportion of recombining genes in each species that are part of the cargo genes of prophages. Of the genes detected by fastGEAR in the pan-genomes of *S. agalactiae, S. pyogenes* and *S. suis*, 553, 914 and 1143 genes were associated with prophages, representing 22.75%, 29.28% and 18.75% of the total number of recombined genes, respectively (Fig. 4B).

In *S. agalactiae*, the recombined genes with known function that were most commonly found in phages include *ftsK_2*, *opuCD*, *pac*, *pre* and *yoeB* (Fig. 4B). The gene *ftsK_2* is involved in the control of recombination machinery in several *Streptococcus* species, including *S. agalactiae*^49^. The gene *pre* codes for a plasmid recombinase in *S. agalactiae*^50^. The gene *pac* codes for a cell-surface antigen and is associated with phagocytosis susceptibility^51^. The genes *opuCD* are part of the *opu* operon encoding for an carnitine transporter and is involved in osmoregulation^52^. Finally, the gene *yoeB* is part of toxin-antitoxin systems of *S. suis* and *S. pneumoniae*, and is implicated in oxidative stress response and biofilm formation^53^*.*

In *S. pyogenes*, the recombined genes most commonly found on phages include *clpP_2*, *lexA*, *xerC* and *xerD* (Fig. 4B). The gene *clpP_2* codes for a subunit of the ATP-dependent protease Clp, which is associated with stress tolerance and virulence during *Streptococcus* infection^54^. The global transcription factor LexA controls DNA repair, but it is also appropriated by mobile genetic elements to enable it to respond to multiple stresses^55^. The genes *xerC* and *xerD* code for site-specific tyrosine recombinases in *Streptococcus*^49^.

In *S. suis*, the recombined genes most commonly found on phages include *ezrA, rodA, serB* and *yajL*, and most of these genes are involved in bacterial growth (Fig. 4B). EzrA is involved in biofilm formation and interspecies competition in *Streptococcus mutans*^56^. RodA is a peptidoglycan polymerase that function in cell growth in *Streptococcus*^57^. The gene *serB* codes for phosphoserine phosphatase, which can induce inflammation and immune response^58^. Finally, the gene *yajL* codes for a glyoxalase in *Escherichia coli* that is used in oxidative stress response^59^.

### Horizontal acquisition of antibiotic resistance and virulence genes via mobile elements

We also sought to identify horizontally acquired resistance genes associated with mobile genetic elements. Here, we focus on integrative and conjugative elements (ICEs), which are autonomously mobile entities that encode their own excision, transfer via conjugation and integration into the same chromosome or in a new host cell^60,61^. We also searched for integrative and mobilizable elements (IMEs), which do not carry their own conjugation machinery^61,62^. Instead, they take advantage of the conjugation machinery of ICEs. ICEs and IMEs can carry cargo genes that confer novel phenotypes to their new host cell, such as antibiotic resistance. Using ICEfinder^61^, we detected 0–8, 0–3 and 0–6 ICEs and IMEs per genome in *S. agalactiae, S. pyogenes* and *S. suis*, respectively (Supplementary Table S6). These numbers were consistent with those previously reported in the three species but which were analyzed using fewer genomes than in our study^63^. We detected numerous cargo genes in the pan-genomes of each species that were associated with ICEs and IMEs, with an average of 39.14, 16.10 and 51.59 cargo genes per genome in *S. agalactiae, S. pyogenes* and *S. suis*, respectively (Fig. 4C). While the identified resistance genes and virulence factors make up only a small proportion of the total ICE/IME-associated genes, their impact on human health merits emphasis. Genes conferring antimicrobial resistance comprised 1.65%, 1.78% and 1.49% of the average ICE/IME-associated cargo genes per genome in *S. agalactiae, S. pyogenes* and *S. suis*, respectively. Virulence genes made up 0.53%, 2.56% and 0.005% of the average ICE/IME-associated cargo genes per genome in *S. agalactiae, S. pyogenes* and *S. suis*, respectively.

## Discussion

In this study, we sought to characterize the genetic diversity and quantify frequencies of within-species recombination in *S. agalactiae, S. pyogenes* and *S. suis*. We used large-scale genomic datasets numbering to more than 1000 genomes per species. Our results showed that recombination represents an important force shaping the evolution and diversity of the three *Streptococcus* species. Previous studies have reported the contributions of recombination in these three species^9,22–25^, and our current study greatly expands on these previous work using the largest genomic datasets to date. This allowed us to investigate in detail the extent of recombination in the core and accessory genomes of each species. A remarkable proportion of each species’ pan-genome have had a history of recombination (12.85%, 24.18% and 20.50% of *S. agalactiae, S. pyogenes and S. suis*, respectively). Whether these recombination events are adaptive or neutral remains to be investigated.

Genetic recombination facilitates access to expansive species gene pools and hence shapes diversification of distinct lineages within each *Streptococcus* species. It also introduces novel allelic variants that may confer unique phenotypes or adaptive characteristics^16,17,64^. This process is therefore particularly useful in organisms inhabiting a wide range of niches and are exposed to selective pressures from different hosts or environmental changes^9,65^. In all three species, a portion of recombined genes have been acquired through mobile genetic elements, indicating that these mobile elements play an important role in facilitating recombination. In *S. pneumoniae*, ICEs and IMEs are important vehicles for the widespread dissemination of mobilizable resistance genes, which can lead to the rapid emergence of multidrug resistant lineages as in the case of the multidrug-resistant pandemic clone Spain 23F ST81^66^. A recent study on the prevalence and diversity of ICEs and IMEs across the *Streptococcus* genus has yielded novel and diverse families of mobilizable proteins^63^, which further highlights the importance of investigating them. Prophages in *Streptococcus* are remarkably diverse and widespread, and have been implicated in pathogenesis and increased infection^67^. We found that many of the recombined genes detected were associated with these phages.

We acknowledge the limitations of our study. First, the datasets we used were composed of publicly available genomic sequences from different sampling sources, research laboratories and geographical regions. This means that certain genotypes, lineages and sequence variants were likely overlooked in our analyses, which can potentially influence our pan-genome and recombination analyses. Because many of the sequences did not include associated metadata, we were unable to examine the impacts of serotypes and ecological sources to recombination frequencies. Notwithstanding these limitations, we obtained sufficient representation of each species to provide an initial genome-wide perspective on the frequencies and characteristics of recombination. Our reliance on existing databases to detect mobile genetic elements and antibiotic resistance genes also limits our ability to discover novel genomic elements that may play an important role in recombination within each species. Second, recombination between closely related strains with near identical DNA sequences is difficult to detect using current methods. Recombination events occurring multiple times on the same chromosomal sites are also challenging to identify. Hence, our results are likely an underestimation of the true extent of recombination found in nature for the three species. Lastly, we acknowledge that different methods to characterize the bacterial pan-genome greatly vary. It is also known that in many bacterial species, the size of the pan-genome is known to be impacted by the number of strains compared, stringency of defining gene occurrence (e.g., a gene present in all of 100 genomes or only 95% of 1000 genomes), and the identity and coverage thresholds used to calculate the gene clusters. In our analysis, we opted to use a 95% cut-off value to define gene clusters and split paralogous genes into different gene clusters. While more work is needed to fine-tune pan-genome estimation methods in the future, our results clearly show large differences in genome-wide recombination frequencies among the three species, which was the goal of this study.

The results presented here open multiple avenues for future research. Future work should focus on the relative contributions of the specific mechanisms (e.g., transduction, conjugation, transformation^68^) for acquiring extrachromosomal DNA among the three species. The roles of other less well known mobile genetic elements in *Streptococcus* and the specific genes they mobilize also need to be explored further. The extent of recombination can also greatly between strains or lineages within each species^69,70^, which may reflect certain ecological determinants (e.g., interaction with the host, antibiotic exposure) driving recombination at the subspecies level. We therefore emphasize the need to include comprehensive associated metadata in sampling and genome sequencing efforts. Distinct patterns of recombination have also been reported between multidrug resistant and hyper-virulent strains of a species, which may also be true in the *Streptococcus* species^71^. DNA can also be acquired from outside of the species as reported in *S. pneumoniae*^72,73^; hence, quantification of recombination events between species or even genera will provide important insights to the frequent donor lineages and the ecological context that enables recombination. Characterizing these patterns will help elucidate measures to control the spread of lineages with clinically relevant features or unpredictable phenotypes. Lastly, in vitro functional assays will help us understand the selective pressures that frequently recombining genes are subject to and their roles in the long-term evolutionary history of each species.

In summary, this study provided detailed insights into the dynamics and extent of homologous recombination and mobilizable species pan-genomes in shaping the diversity of *S. agalactiae, S. pyogenes* and *S. suis*. These mechanisms enable the creation of novel combinations of genes and sequence variants that selection can act on and the potential for high-risk clones to emerge. Continuous surveillance of hyper-recombining lineages and/or frequently recombining genes will inform future approaches to disease control and transmission caused by these species.

## Methods

### Dataset

Genome assemblies were downloaded from the NCBI RefSeq^26^ database in May 2019. We included all named genomes that were available at that time. Unfortunately, associated metadata were lacking for many of these genomes. The genomes were annotated using Prokka v.1.13.3 with default parameters^74^. To determine the degree of genomic relatedness and hence clarify whether these genomes belong to the same species, we calculated the genome-wide average nucleotide identity (ANI) for all possible pairs of genomes within each species using the program FastANI v.1.1^75^. We used the threshold value of 95% as a cutoff to define whether strains belong to the same or different species^75^. Among the *S. agalactiae* genomes, a divergent strain with < 80% identity with the other strains was removed from further analysis. A BLAST^76^ search of the 16S rRNA gene of the divergent strain against NCBI non-redundant sequence database showed that it was most similar to *S. hyovaginalis*. All the *S. pyogenes* genomes fell within ≥ 95% identity value. The *S. suis* genomes had a divergent cluster with < 90% identity with the rest of the strains, and these genomes were also subsequently removed from further analysis. We used the R v.3.6.3^77^ package gplots^68^ to build an heatmap of pairwise ANI. In all, we compiled a total of 1081 *S. agalactiae*, 1813 *S. pyogenes* and 1204 *S. suis* genomes that we used for all downstream analyses.

### Pan-genome and phylogenetic analyses

For each species, we identified the core and accessory genes using Roary v.3.12.0 with default settings^78^. Roary iteratively pre-clusters protein sequences using CD-HIT^79^, which results in a substantially reduced set of data. Sequences in this reduced dataset were then compared using all-against-all BLASTP^76^ and were clustered the second time using Markov clustering (MCL)^80^. By default, Roary uses the conserved gene neighborhood information to split homologous groups containing paralogs into the most appropriate cluster based on synteny. Any cluster containing paralogous genes is filtered out of the final core gene alignment. We opted to split paralogous genes because they may lead to inaccurate interpretations in a core-genome phylogenetic and mcorr analyses. We also used the default 95% minimum identity for sequence comparisons. Minimum identity cut-off values ranging from 75 to 100% in increments of 5% were also tested (Supplementary Figure S1; Table S1), but the downstream analyses were performed only with the 95% cut-off value pan-genome. Each orthologous gene family from the merged CD-HIT and MCL was aligned using MAFFT^81^. We generated the plots summarizing the pan-genome data using modified versions of the scripts create_pan_genome_plots.R and roary_plots.py available in Roary^78^. The sequence alignments of each identified core gene family were concatenated to give a single core genome alignment. We used this core genome alignment to build a maximum likelihood phylogeny using the program RAxML v.8.2.11^82^ in rapid hill-climbing mode with a general time reversible (GTR) nucleotide substitution model^83^ and four gamma categories for rate heterogeneity. We also used the core genome alignments to delineate distinct clusters or sub-populations using RhierBAPS^28^. RhierBAPS is an R^77^ implementation of the clustering algorithm hierBAPS (hierarchical Bayesian Analysis of Population Structure)^84^, which estimates the hierarchical clustering of DNA sequence data to reveal nested genetic population structures^28^. We also used PopPUNK v.2.4.0 to delineate clusters based on shared sequence and gene content distances^85^. Database sketches were created using values of *k* ranging from 15 to 29 in increments of 2 for *S. agalactiae* and *S. pyogenes*, but a database could not be sketched successfully from the *S. suis* population. For assigning queries, four clusters were detected in *S. pyogenes*, while no clusters were found in *S. agalactiae*. The RhierBAPS clusters were therefore used for comparisons between species. The sequence type (ST) of each isolate was confirmed using the program MLST v.2.19.0 (https://github.com/tseemann/mlst), which extracts seven housekeeping genes from the sequence contigs and compares sequence variation against previously characterized STs in the PubMLST database^86^.

### Mobile element detection

We used ICEfinder v.1.0 and the ICEberg 2.0 database^61^ to identify ICEs and IMEs. ICEfinder first detects recombination and conjugation modules in the target genome using profile Hidden Markov Model (HMM). It then looks for the *oriT* region and performs pattern-based co-localization filtering of the genes. We identified the most commonly occurring genes in ICEs and IMEs across all three species and plotted a histogram of these using R v.3.6.3^77^. We also used ICEfinder to identify virulence genes and antibiotic resistance genes associated with ICEs and IMEs^61^. Next, we used PhiSpy v.4.2.19 with default settings to identify prophages^48^. PhiSpy detects prophages using both similarity-independent (protein length, transcription strand directionality, AT and GC skew, and phage DNA sequence word abundance) and similarity-based measures (phage insertion points and phage protein similarity)^48^. Graphical visualization of the number of ICEs, IMEs, prophages, and cargo genes was done using the R v.3.6.3^77^ package ggplot2^87^.

### Recombination detection

We used two methods to detect recombination. Mcorr estimates recombination rates using the core genome alignment, while fastGEAR identifies recombinant sequences in core and shared accessory genes. We considered the results of these two methods separately.

First, we used the program mcorr with default parameters to compare correlated synonymous substitutions for each pair of homologous sequences^30^. These correlated substitutions were then used to estimate different evolutionary and recombination parameters: mutational divergence, recombinational divergence, recombination coverage or proportion of sites in the genome whose diversity was derived from outside the sample through recombination, mean recombination fragment size, diversity, and relative rate of recombination to mutation^30^. The core genome alignment of each species was used as input in mcorr with 1000 bootstrapped replicates. Next, we ran fastGEAR with default parameters to identify specific core and accessory genes that have had experienced recombination^33^. FastGEAR detects recombinations by first differentiating the gene sequences into lineages (or clusters) and then compares every nucleotide site in a target sequence to other members of its own lineages and to other lineages^33^. FastGEAR uses gene-by-gene alignments to determine lineages of each gene, and the cutoff for determining lineages corresponds to similarity across 50% of the sequence length. The cutoff for detecting recombination events is an HMM-based probability of the assigned lineage being less than 0.05. We ran a diversity test implemented in fastGEAR to test the significance of the inferred recombinations and identify false-positive recombinations. The recombination events of each gene were plotted using R v.3.6.3^77^.

Unless otherwise indicated, we used default parameters for the programs we used, following previous population genomic studies of *Streptococcus*^9,25,47^.

## Supplementary Information

Supplementary Table S1.

Supplementary Table S2.

Supplementary Table S3.

Supplementary Table S4.

Supplementary Table S5.

Supplementary Table S6.

Supplementary Table Legends.

## Data Availability

The datasets analyzed in this study were downloaded from and are available in the GenBank database (https://www.ncbi.nlm.nih.gov/genbank/). Accession numbers are listed in Supplementary Table S1.
