# Supplementary material for: Frequencies and characteristics of genome-wide recombination in Streptococcus agalactiae, Streptococcus pyogenes, and Streptococcus suis
Source: Sci Rep. 2022 Jan 27;12:1515. doi: 10.1038/s41598-022-04995-5 (PMC8795270; doi:10.1038/s41598-022-04995-5)
Supplement: Supplementary file 7 — Supplementary Table Legends. [file 41598_2022_4995_MOESM7_ESM.pdf]

Supplementary information for Frequencies and characteristics of genome-wide recombination in *Streptococcus agalactiae*, *Streptococcus pyogenes*, and *Streptococcus suis*

Isaiah Paolo A. Lee [a] and Cheryl P. Andam [b]

[a] University of New Hampshire, Durham, New Hampshire 03824 USA
[b] University at Albany, State University of New York, New York 12222 USA

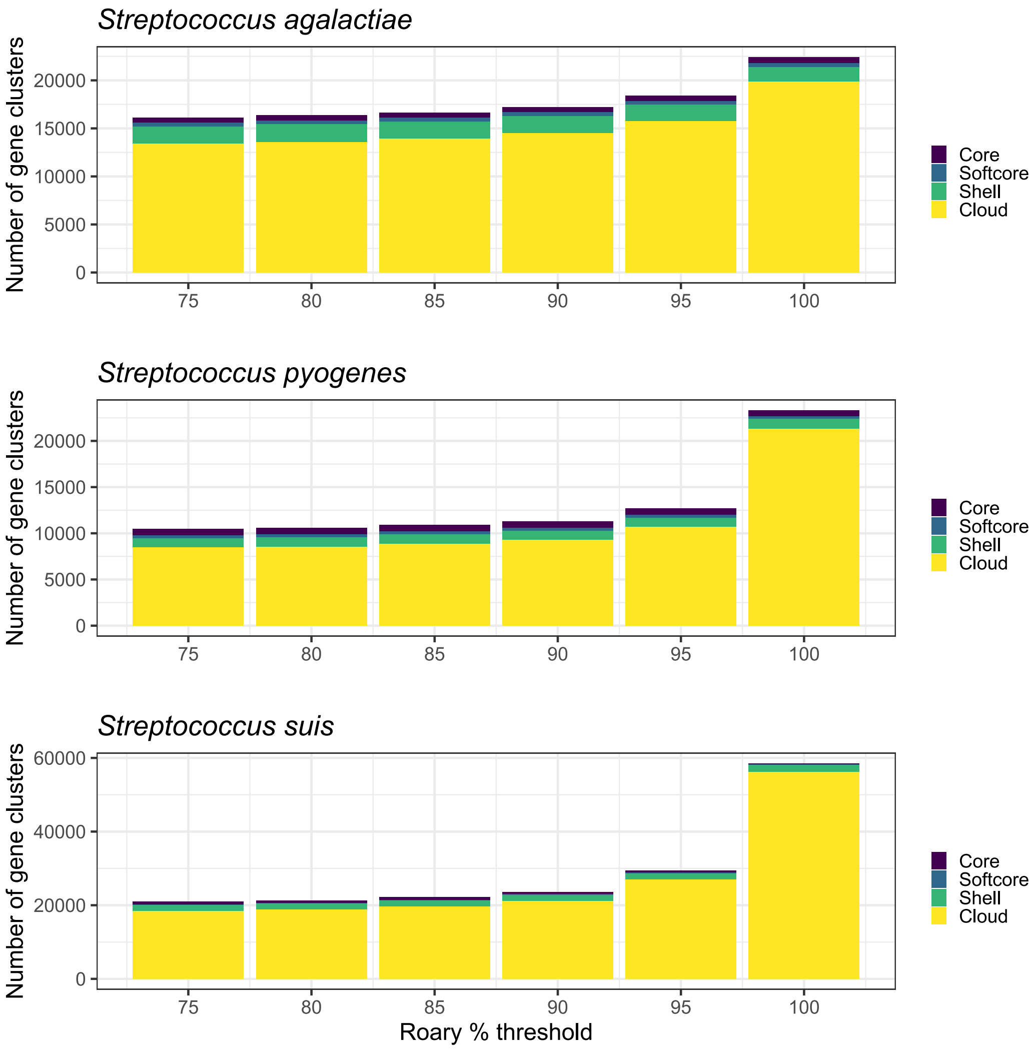

Supplementary Figure S1. Comparison of cut-off values used in Roary to define gene clusters

Supplementary Table S1. Accession numbers, genomic characteristics and MLST of genomes used in this study (different tabs)

Supplementary Table S2. Results of the Roary analysis for *S. agalactiae, S. pyogenes* and *S. suis.* Comparison of the different cut-off values to defining gene clusters is also shown (different tabs)

Supplementary Table S3. Results of the mcorr analysis for *S. agalactiae, S. pyogenes* and *S. suis* (different tabs)

Supplementary Table S4. Results of the fastGEAR analysis for *S. agalactiae, S. pyogenes* and *S. suis* (different tabs)

Supplementary Table S5. Results of the PhiSpy analysis for *S. agalactiae, S. pyogenes* and *S. suis* (different tabs)

Supplementary Table S6. Results of the ICEfinder analysis for *S. agalactiae, S. pyogenes* and *S. suis* (different tabs)
